# A Comprehensive Study on the Antimicrobial Properties of Resveratrol as an Alternative Therapy

**DOI:** 10.1155/2021/8866311

**Published:** 2021-03-16

**Authors:** Ehsan Abedini, Ehsaneh Khodadadi, Elham Zeinalzadeh, Seyyed Reza Moaddab, Mohammad Asgharzadeh, Bahareh Mehramouz, Sounkalo Dao, Hossein Samadi Kafil

**Affiliations:** ^1^Drug Applied Research Center, Faculty of Medicine, Tabriz University of Medical Sciences, Tabriz, Iran; ^2^Hematology and Oncology Research Center, Tabriz University of Medical Sciences, Tabriz, Iran; ^3^Pharmaceutical Nanotechnology Research Center, Tabriz University of Medical Sciences, Tabriz, Iran; ^4^Biotechnology Research Center, Tabriz University of Medical Sciences, Tabriz, Iran; ^5^Connective Tissue Diseases Research Center, Tabriz University of Medical Sciences, Tabriz, Iran; ^6^Faculté de Médecine, de Pharmacie et d'Odonto-Stomatologie (FMPOS), University of Bamako, Bamako, Mali

## Abstract

Resveratrol is a polyphenolic antioxidant whose possible health benefits include anticarcinogenic, antiaging, and antimicrobial properties that have gained significant attention. The compound is well accepted by individuals and has been commonly used as a nutraceutical in recent decades. Its widespread usage makes it essential to study as a single agent as well as in combination with traditional prescription antibiotics as regards to antimicrobial properties. Resveratrol demonstrates the action of antimicrobials against a remarkable bacterial diversity, viruses, and fungus. This report explains resveratrol as an all-natural antimicrobial representative. It may modify the bacterial virulence qualities resulting in decreased toxic substance production, biofilm inhibition, motility reduction, and quorum sensing disturbance. Moreover, in conjunction with standard antibiotics, resveratrol improves aminoglycoside efficacy versus *Staphylococcus aureus*, while it antagonizes the deadly function of fluoroquinolones against *S. aureus* and also *Escherichia coli*. The present study aimed to thoroughly review and study the antimicrobial potency of resveratrol, expected to help researchers pave the way for solving antimicrobial resistance.

## 1. Introduction

One of the significant causes of death and morbidity worldwide is infectious diseases [1]. The therapy of many bacterial infections becomes much more troublesome and leads to a major challenge due to the rising incidence of antibiotic resistance and shortage of adequate drug choices [2, 3]. Moreover, the absence of antibiotics, inappropriate use in health treatment, and alteration in some genes is another major problem [4, 5]. Antibiotics are usually indiscriminate in treating bacterial infections although unavoidable adverse results such as nausea, diarrhea, allergic reactions, and drug interactions are possible [6]. There is a robust demand for other microbicides to monitor disorders owning to the quick spread of antibiotic resistance [7]. Researchers are actively seeking unique antibiotics with efficacy effects and the lowest adverse reactions. One of the critical answers for the problems stated above is natural antibiotics [8, 9]. Therefore, botanical antimicrobials can be useful among natural antibiotics to treat Gram-positive as well as damaging microbes [10].

Resveratrol (RSV) has been the first isolated stilbenoid substance characterized by the white hellebore base, *Veratrum grandiflorum* [11, 12]. As with another member of the stilbene group, the development of RSV is triggered by pathogen reaction, ultraviolet irradiation, or direct ozone exposure [13, 14]. This substance is obtained clearly from different plant varieties, such as grapevines, pines, bananas, beans, and even high amounts of pomegranates, peanuts, and soybeans [15–17]. RSV has been identified as playing a possible role in body immune system control and inflammation, chemoprevention, neuroprotection, cardioprotection, lipid control, and treating diseases such as diabetic issues, Parkinson's, and cancer. Furthermore, RSV demonstrated antibacterial, antiviral, and also, antifungal function [18, 19]. As a natural substance, thus, emphasis on the impact of RSV on antimicrobials can be rewarding.

Recently, RSV has been a big issue owing to its possible beneficial effects [20]. An example can be the “French paradox.” The consumption of red wine, with a high RSV content, was related to low cardiovascular mortality in the French people, given their high cholesterol levels [21, 22]. RSV has been investigated for its antibacterial activity against pathogenic organisms, in addition to detailed work on a multitude of diseases. This study concentrated on RSV's antimicrobial properties. This study emphasized the antiviral functions of RSV in animal and human bacterial infections and how these kinds of these results are connected with the compound's antioxidant activities.

## 2. Structure and Plant Sources of Resveratrol

RSV (3, 5, 4′-trihydroxystilbene) is a natural polyphenolic antioxidant in the stilbene family. The stilbene family includes a C6–C2–C6 carbon skeleton (1,2- diphenylethylene), in which RSV can be a hydroxylated derivative [23]. RSV is found in several plants, including peanuts (*Arachis hypogea*), blueberries and cranberries (*Vaccinium* spp.), Japanese knotweed (*Polygonum cuspidatum*), a traditional Asian herbal medicine, and most notably, grapevines (*Vitis vinifera*), as an all-natural supply for human consumption [24]. Plants synthesize RSV as a potential phytoalexin in reaction to microbial pathogens damage and UV radiation [25, 26]. In noninfected grapes surrounding grapes defecated with an excrete, RSV synthesis is most prominent in preventing the fungi spread to healthy and balanced grapes, as shown by the fungi *Botrytis cinerea*, the necessary trans agent of gray mold [27–29]. RSV occurs in both a *cis* as well as a *trans* isomer [30], while the *trans* isomer seems to be expected in wine form [31] and the best analyzed because of its better quantity as well as durability [32]. The RSV content of red wine is typically higher than that of white wine [33]. The typical red wine contains 1.9 mg/L of *trans-*RSV, although it can exceed 14.3 mg/L in some situations [34].

## 3. Chemical Synthesis of Resveratrol

Since the focus of natural RSV in plants is restricted and the extraction is expensive, many chemical synthesis and biosynthesis efforts have been made.

The Heck reaction is the C–C coupling response of an aryl halide and perhaps a vinyl halide with a triggered olefin following the catalysis of palladium in the existence of a base [35]. Current growths in reaction mechanisms have revealed various donors and acceptors appropriate for Heck reactions [36]. A variety of Pd catalysts immobilized onto heterogeneous assistances are applied to synthesize pterostilbene [37]. Considered that retrosynthesis approaches can be employed to evaluate RSV derivatives' compounds, they can be used to synthesize essential polyphenolic substances [38]. The Heck–Mizoroki C-C cross-coupling reaction can be a crucial stage that is virtually enhanced by palladium nanoparticles sustained on synthetic clay. In this response, the catalyst presents high effectiveness and also is conveniently managed. The trigger can be recuperated and recycled numerous times. Besides, the purification phase utilizes minimal solvents [39]. RSV is a phytoalexin that can be acquired by the decarbonylative Heck reaction. In this reaction, 3,5-dihydroxybenzoic acid was combined with 4-acetoxy styrene in the existence of palladium acetate and N, N-bis-(2,6-diisopropyl phenol) dihydro imidazolium chloride to synthesize RSV derivatives [40]. A technique based upon the palladium-catalyzed oxidative Heck reaction that applies boronic acid and styrene as catalysts was also established to synthesize resveratrol. RSV was synthesized by the Heck reaction and chosen differently in vitro and cell-based targets to identify RSV activity related to sulfate metabolites [41, 42].

The Perkin reaction is another organic response for converting aromatic aldehydes and anhydrides right into alpha- and beta-unsaturated carboxylic acids by sodium acetate, base, and acid therapy. The response includes defense, condensation, decarboxylation, and deprotection [43]. The last response item can be regioselective. The Perkin reaction with p-anisyl acetic acid sodium salt and 1,3-dimethoxy benzaldehyde as the catalysts in the existence of acetic anhydride to synthesize RSV was employed [44]. The decarboxylation with quinolone–Cu salt created the RSV derivative's final product, which has a framework similar to the all-natural item [45].

The Wittig reaction uses the reformation of main alkyl halide and aldehyde/ketone to generate an olefin element under triphenylphosphine activity and a base to free a triphenylphosphine-oxide by-product [46]. This reaction was generally employed to create a C–C double bond [47]. Moreover, to synthesize RSV and its analogs in benzyl alcohols as phosphorus ylide companions, a one-pot Wittig-type olefination reaction was used [48].

In addition to normal RSV reactions, another method for preparing RSV and its derivatives was discovered. RSV and its products were synthesized via the Horner–Wadsworth–Emmons reaction, and it was found that certain substances had a discerning capacity to inhibit cyclooxygenase-1 and cyclooxygenase-2 [49]. RSV from grapevine leaves was obtained by aluminum chloride induction. This report offers convincing proof that metallic salt can directly generate phytoalexin response. This technique can also be used to monitor *Botrytis cinérea* in vineyards [50]. Researchers have established previous biosynthesis and biomimetic synthesis methods for RSV and its analogs [51]. Likewise, scholars performed *Agrobacterium-tumefaciens*-mediated transformation into the apple and observed that it could produce RSV with stable inheritance [52].

## 4. Potential Shortcomings of Resveratrol

RSV has low water solubility and inadequate oral bioavailability and is quickly metabolized in the system. Its low bioavailability is attributed to its fast metabolism in the liver into glucuronides and sulfates [53]. Although the quantity of oral dosing of RSV did not notably influence its bioavailability in plasma, the kind of food taken in and intraindividual distinctions in metabolism were revealed to affect its bioavailability [54] considerably. In a research study, the plasma bioavailability of RSV 30 minutes after oral red wine intake was only in trace quantities. In contrast, minutes later, RSV glucuronides were systemically plentiful for a prolonged time [54, 55]. Lately, another study has been concentrated on emerging structured nanoparticles that will undoubtedly improve the bioavailability of RSV and prolong its launch *in vivo*. Solid lipid nanoparticles (SLNs) and nanostructured lipid carriers (NLCs) filled with RSV were revealed to have an entrapment efficiency of 70% and stability lasting for over two months. *In vitro* simulation reports displayed a slow, continued release of RSV at both stomach and intestinal pH levels [56]. Likewise, the use of zein-based nanoparticles was demonstrated to affect the *in vivo* delivery of RSV in a mouse model of endotoxic shock [57].

In spite of the lipophilic features that enable its absorption, RSV displays a low bioavailability that, by decreasing its efficiency *in vivo*, it can be an obstacle for development in new treatments [58, 59]. Estimates of the plasmatic focuses of RSV metabolites, following oral administration, are notably higher than totally-free RSV, showing a high-speed rate of RSV metabolism (a half-life 0.13 h) [17], reaching plasmatic ranks between 0.3 and 2.4 *μ*M [60]. Furthermore, the experimental finding has recommended that the matrix's function (e.g., in alcohol, natural sources, and vehicles) provides RSV in humans, causing notable variability [53]. This eventually results in the delivery of percentages of free RSV in the plasma and, consequently, in the cells [61]. The organic activities of the conjugated metabolites and the conversion of both sulfates and glucuronides to RSV, particularly organs such as the liver, may facilitate drug effectiveness and bioavailability [62].

Nonetheless, the content of RSV in the human regimen is almost small. Therefore, manufacturers have attempted to maximize the drug's beneficial properties by selling RSV in supplementary pills and liquids. It is often integrated with vitamins or other compounds [63].

## 5. Potential Therapeutic Usage of Resveratrol

RSV has been reported with extensive medicinal uses and health benefits. This can also be used as a scaffold to design systemic relationships that can potentially mediate more extreme responses with enhanced mechanistic stringency [64, 65]. RSV plays a vital role in mitigating the symptoms of several disorders, such as diabetes, cancer, and Parkinson's disease, via its anti-inflammatory, antioxidant, antiproliferative, and antiangiogenic properties [66, 67]. Furthermore, RSV has been studied as an anticancer natural product by *in vitro* and *in vivo* inhibition of tumor cell lines including myeloma, breast cancer, lymphoma, colorectal cancer, melanoma, and hepatocellular, pancreatic, and prostate carcinoma [68]. The latest findings have demonstrated the impact of RSV on signaling molecules and downregulation of angiogenesis-associated gene expression [68, 69], cell cycle arrest activation [70], and apoptosis stimulation.

On the other hand, RSV makes drugs such as paclitaxel- [71], thalidomide-, and Velcade-resistant tumor cell lines [72]. The appearance of antimicrobial resistance amongst microbial agents is unavoidable, and new approaches for controlling resistant pathogens are needed. Because of their medical benefits and lack of adverse effects [73], natural products' use has grown in considerable popularity among research groups.

## 6. Mechanism of Resveratrol Action

RSV has been revealed to cause apoptosis by downregulating and upregulating various genes significant in cell functions and, however, not restricted to TRAIL-R2/DR5, TRAIL-R2/DR4, p53, Bim, Noxa, PUMA, Bak, Bax, Mcl-1, survivin, Bcl-XL, and Bcl-2. RSV has been revealed to prevent cellular development at G1 and G1/S stages and be an anti-inflammatory mediator by restraining nuclear factor-kappa *β* (NF-*κβ*) activity, procyclooxygenase- 2 activity, and prostaglandin production [74]. Moreover, its activities in postponing the start of heart diseases and cancer cell progression, in addition to antiviral effects, have also been commonly studied [75].

The notable beneficial impact of *trans*-RSV is connected with antioxidant, antiproliferative, and anti-inflammatory properties [76]. RSV is renowned for hindering platelet activation and oxidation of low-density lipoproteins and decreasing the intracellular development of peroxide and superoxide radicals in skin fibroblasts *in vitro* [77]. The chemopreventive results of RSV are connected to quinone reductase 2, which subsequently boosts cell antioxidants' expression and detoxifying enzymes to increase cellular resistance to oxidative stress [78].

Its anti-inflammatory endeavor is caused by inhibition of cyclooxygenase 1 *in vitro* and cyclooxygenase 2 in mouse skin [79]. The antioxidant activity of RSV may be associated with some of its functions as a possible scavenger of peroxyl and superoxide radicals or with its capability to reduce oxidation through inhibiting the enzyme [80]. *Trans*-RSV has been discovered to function as a much better radical scavenger than vitamins E and C. Nevertheless, an upgraded synergistic result is attained in RSV with one of the vitamins [81]. The growth of reactive oxygen species in the skin leads to various skin diseases, such as cancer [82].

Chronic UV radiation exposure, which can trigger DNA damages, is an additional significant issue in cutaneous disorders' pathogenesis [83]. Chemoprevention refers to the use of agents that may inhibit, reverse, or delay the influence of UV radiation exposure to the skin [84]. Survivin, a participant of the inhibitor of the apoptosis gene family and vital regulator of survival or death of cells, plays a role in the pathogenesis of sunlight-induced cancer cells in addition to chromosomal modifications and mutations [85]. RSV might guarantee skin protection versus UVB-mediated serious harms and future cancerogenic growth by modulating survivin's expression and activity [86]. Additionally, RSV might be applied in the adjunctive treatment of melanoma [87]. It decreases the possibility of melanoma cells and surges the cytotoxicity of temozolomide on malignant cells. Furthermore, RSV can hinder redox factor 1, making melanoma cells much more sensitive to radiation treatment [88].

## 7. Antimicrobial Properties of Resveratrol

Antibacterial therapy is an effective method for treating various diseases and is a crucial element of the contemporary medical approach [26]. Nevertheless, the boosted resistance of microorganisms to the antimicrobials commonly used has contributed to evaluating many agents with possible antimicrobial activity [89].

An antimicrobial agent has significantly decreased the risks related to infectious diseases over the last century [90]. Incorporated with signs of progress in sanitation, healthcare, and nutrition, as well as the implementation of robust immunization programs, the use of these medications has facilitated a dramatic reduction in untreatable, sometimes lethal, infectious diseases, leading to an improved life expectancy [91]. However, adapting microorganisms' defenses versus the antibiotics applied has made the growth, proliferation, and determination of antimicrobial resistance a significant public health concern at the moment [46].

It is important to remember that, in addition to discover new antibiotics to be used as medicines, the production of new preservatives in the food industry often requires a large amount of work [92]. Therefore, while most synthetic preservatives are successful, consumers are more concerned about their health, indicating a rising concentration in new antimicrobial substances acquired from all-natural resources [91]. There has been a significant focus on biologically active compounds and other synthetic bases [93].

RSV has, thus, been the main topic of examination, in addition to the biological activities mentioned above, for its capability to restrain the development of certain pathogenic microorganisms, such as Gram-positive and Gram-negative bacteria, as well as fungi [94, 95]. Hence, RSV has potential because of its antimicrobial properties and can be applied to treat and prevent infections triggered by specific pathogens in the future, according to these findings (Table 1).

## 8. Antifungal Activity

RSV usually revealed far better antifungal than antibacterial activity, as displayed by the minimum inhibitory concentrations (MICs). RSV inhibitory activity is about 25–50 *μ*g/mL for the fungal dermatophytes *Trichophyton mentagrophytes*, *Trichophyton tonsurans*, *Tri-chophyton rubrum*, *Epidermophyton floccosum*, and *Microsporum gypseum* [26]. The inhibitory action for the fungal species *Candida albicans*, *Saccharomyces cerevisiae*, and *Trichosporon beigelii* is 10–20 *μ*g/mL [112], although another study has not identified antifungal activity against *C. albicans* [105, 106]. RSV activity was inhibitory against plant pathogen *B. cinerea*, the whole organism of gray mold in which B germination has been developing. Concentrations of 60–140 *μ*g/mL are observed for *B. cinerea* conidia and mycelial development [107].

## 9. Antiparasitic Activity

This polyphenol compound's antiparasitic activity was measured against *Trypanosoma cruzi*, *Setaria cervi*, and *Leishmania amazonensis* [113]. In *Leishmania amazonensism*, RSV exhibited antipromastigote and antiamastigote effects, boosted promastigote proportion, decreased mitochondrial capacity, and decreased arginase enzyme activity of macrophages leading to the removal of parasites [114]. The findings of other studies showed that *trans*-RSV analogs have activity in anti-*L. amazonensis* and cause adventitious death by promastigotes [115]. In the filarial nematode, *Setaria cervi trans*-stilbene derivatives have exercised anti-filarial activity through reactive oxygen species (ROS) generation and apoptosis mediation [116]. RSV exposed strong antiparasitic effects on *T. cruzi* through supported metacyclogenesis, inhibiting epimastigotes growth, blocking differentiation, and replicating intracellular amastigotes [109].

## 10. Antibacterial Activity

RSV inhibits development at concentrations <100 *μ*g/mL for a small variety of bacterial species, including the *Bacillus cereus* (MIC = 50 *μ*g/mL) [104], *M. Smegmatis* (MIC = 64 *μ*g/mL) [100], *Helicobacter pylori* (MIC = 25–50 *μ*g/mL) [117], *Vibrio cholerae* (MIC = 60 *μ*g/mL) [110], *Neisseria gonorrhoeae* (MIC = 75 *μ*g/mL) [26, 118], *Campylobacter coli* (MIC = 50 *μ*g/mL) [119], and *Arcobacter cryaerophilus* (MIC = 50 *μ*g/mL) [96], respectively. Resveratrol's inhibitive activity versus *Mycobacterium tuberculosis* is 100 *μ*g/mL [97].

RSV only exhibits growth-inhibitory behavior at concentrations >100 *μ*g/mL for many bacterial organisms. Remarkable Gram-positive pathogens with MICs of about 100–200 *μ*g/mL contain *S. Enterococcus faecalis* [98, 99] and *Streptococcus pyogenes* [26, 118]. Some studies recorded a lower sensitivity to multiple Gram-negative pathogens (MIC > 200 *μ*g/mL) relative to Gram-positive pathogens, such as *E. coli* [120, 121], *Pseudomonas aeruginosa* [101], *Klebsiella pneumoniae* [108], and *Salmonella enterica* serovar Typhimurium [102]. This finding may result from weak RSV penetration of certain Gram-negative bacteria across the outer membrane or maybe the consequence of RSV's active extrusion by efflux pump systems [122]. As RSV inhibits ATP synthase in other bacterial organisms, it remains to be investigated whether diverse bacterial energy generation needs partial accounting for increased RSV susceptibility rates (Table 2).

There are some significant differences between MICs recorded, e.g., for *S. aureus ATCC 25923*, MIC was identified in one study as 100 *μ*g/mL [104] but in other studies as > 1000 *μ*g/mL [111]. One reason for such variability might be variations in the growth medium (Mueller–Hinton and Luria–Bertani, respectively). Still, additional research is needed in generally contradictory outcomes on RSV susceptibility between experiments.

## 11. Antivirulence Properties

Virulence has become a pathogen's capacity to induce infection in a host, and virulence factors are mechanisms by which the pathogen damages the host (e.g., excretion of toxins) along with tools for condition (e.g., adhesion, invasion, invasion, and formation of biofilms) [126]. Virulence gene expression is also tightly controlled for timely and organized environmental adaptation, that is, by quorum sensing (QS) or two-component systems (TCSs) [127, 128]. Antivirulence molecules' therapeutic applicability concerns the reason for disarming the pathogen of capacity to provoke the host's damage and, depending on the host immune system, to kill the bacteria [129] (Table 3).

### 11.1. Antibiofilm Properties

Bacteria can live as planktonic cells or in aggregates attached to surfaces, growing in biofilms as an extracellular material [141]. The benefit of bacteria-based biofilm formation is developing a more stable atmosphere to protect against environmental threats, namely, phagocytosis and antimicrobials [134]. Biofilms have become a clinically significant problem linked to chronic and persistent infections [142, 143].

RSV has also been observed for its capacity to minimize the development of biofilms on different bacterial pathogens [144]. For the Gram-negative anaerobic bacterium *Fusobacterium nucleatum*, involved in the dental plaque, RSV inhibits biofilms' growth in concentrations (4–64-fold below the MIC) that do not affect planktonic cell development [124]. RSV also shows antibiofilm properties against Gram-negative pathogens *V. cholerae* at concentrations 2–6-fold listed below MIC [110] and *E. coli* [103] and the Gram-positive bacterium *P. acnes* [131]. In *E. coli*, the effect is mediated by decreased gene expression (*csgA* and *csgB*) encoding for curli development, which is essential for biofilm formation [103, 130]. For the Gram-positive pathogen *S. aureus*, RSV inhibits the growth of biofilms at concentrations 3-4-fold below the MIC. In combination with vancomycin, RSV has a significant effect on eradicating existing biofilms [132]. Moreover, RSV did not increase biofilm formation in *S. aureus* in two other tests [98, 133], suggesting that the effect may be affected by test conditions and strain variations.

### 11.2. Antimotility Properties

Motility at the colonization stage is essential for several bacterial species [145]. For example, motion can occur by swimming and swarming, involving the development of functional flagella and twitching involving type IV pili [146]. *P. mirabilis* displays decreased swarming capacity in a dose-dependent manner at subinhibitory concentrations of RSV [135]. Swarming suppression in RSV's existence depends on the TCS protein *RsbA*, which is a negative swarming regulator [147]. RSV restricts swimming and swarming at *E. coli* by downregulation with some motility and flagellar genes [26]. *Vibrio vulnificus* has also been reported to have reduced swarming capability [136].

### 11.3. Toxin Interference

Bacterial pathogens contain many structurally and functionally distinct toxins and are, therefore, very significant in disease progress [148]. Surprisingly enough, some studies indicate that RSV interferes with toxins' expression [149]. In *V. vulnificus*, *RtxA1* is an essential multifunctional cytotoxic toxin for lethality in mice, and the treatment with RSV decreases the expression of *rtxA1* [136]. In *V. cholerae*, RSV prevents endocytosis of cholera toxin (CT) into host cells and also binds CT explicitly, possibly inhibiting CT-induced diarrhea [140]. Therefore, RSV significantly reduces *S. aureus* in human blood cells; however, the inhibition process stays mysterious [92, 133].

### 11.4. Interference with Quorum Sensing

Quorum sensing systems allow bacteria to react to density and regulate gene expression through cell-cell communications [150]. Among bacterial pathogens, QS also regulates the virulence gene expression, allowing for a concerted attack that could overpower host defenses [151]. QS involves generating and releasing signal molecules, called autoinducers, which enhance the cells' density [152]. The bacteria detect a threshold limit of the autoinducer, leading to gene expression changes [153]. RSV inhibits the synthesis of the autoinducers *N*-*acyl*-homoserine lactones in *Yersinia enterocolitica and Erwinia carotovora* at a concentration that does not influence growth parameters [137, 138]. RSV affects QS systems in *E. coli* [154] and *Chromobacterium violaceum* [139] via the uncharacterized method. Hence, RSV affects numerous virulence traits at concentrations up to 64 times lower than growth-inhibitory concentrations. If RSV can have some uses as a compound for antivirulence needs to be tested in appropriate animal studies.

## 12. Resveratrol in Combination with Conventional Antimicrobials

Along with acting alone as an antimicrobial agent, RSV combined with traditional antibiotics has also been studied for possible effects. For *E. coli*, RSV (at 0.5 MIC) antagonizes ciprofloxacin's bactericidal function, kanamycin, oxolinic acid moxifloxacin, although the lethality of oxacillin is not affected [155]. For *S. aureus*, the destructive action of daptomycin, moxifloxacin, oxacillin, and levofloxacin is antagonized by RSV [156]. The antagonism mechanism is indicated to include a reduction of RSV in the ROS, which also has antioxidant properties and, thereby, protects macromolecules from ROS damage [155]. ROS production has been implied as contributing to the lethality of bactericidal antibiotics and RSV that suppress bacterial killing with the antibiotics mentioned by scavenging ROS [155, 157].

On the other hand, RSV (at 0.5 regardless of MIC) potentiates the potency of aminoglycosides around 32-fold against *S. aureus* and to a lesser degree against other Gram-positive pathogens such as *S. epidermidis*, *Enterococcus faecium*, and *E. faecalis* [123]. The potentiation mechanism was hypothesized to occur through ATP synthase inhibition as inactivation of ATP synthase encoding genes in *S. aureus* also detects aminoglycosides of this pathogen [158, 159]. RSV further enhances the aminoglycoside activity against *P. aeruginosa*-produced biofilms, but RSV combinations and four separate aminoglycosides did not demonstrate synergies on planktonic cells [160]. Therefore, RSV interferes with various types of antibiotics' inhibitory function. It remains to be investigated if these results are apparent in animal models.

## 13. Potential Antiviral Activity of Resveratrol against Respiratory Viruses

Remarkably, RSV has shown an extensively reported inhibitory activity against viral replication and virus-induced inflammation in infections triggered by pathogenic severe human viruses, including respiratory viruses such as influenza virus, RSV, coronavirus (HCoV), and rhinovirus (HRV) [161].

### 13.1. Influenza Virus

RSV's antiviral activity was revealed in many *in vitro* experiments, evidencing several molecular and cellular mechanisms. RSV treatment effectively prevented dose-dependent replication of the influenza virus (10–20 *μ*g/mL), decreased translation of late viral proteins, and blocked nuclear-cytoplasmic translocation of viral RNPs, a crucial phase in viral replication before virion assembly and release. The inhibition of intracellular signaling pathways, such as protein kinase C (PKC) and mitogen-activated protein kinase (MAPK), has mediated these effects [162]. Subsequently, it was also revealed that an RSV analog restored the host-cell redox imbalance in a dose-dependent manner (5–20 *μ*g/mL), triggered by the virus-induced depletion of GSH levels, which impeded hemagglutinin maturing [163].

Other experimental studies have confirmed RSV's antiviral activity and other stilbene-class compounds against various influenza virus subtypes by inhibiting neuraminidases [164]. Natural stilbenoids isolated from plants, such as *Gnetum pendulum*, affected H1N1 and H3N2 influenza A viruses (inhibitory concentration: 45 *μ*M; toxic dose for 50 percent cell death: 90 *μ*M; therapeutic index: 2) [165]. Ironically, RSV has also been able to prevent the transmission of both human influenza B and swine influenza A viruses [166].

In addition to the direct restraint of virus replication (IC50: 24.7 *μ*M; average development inhibition of 50%: >100 *μ*M; therapeutic index: 4), lately, RSV was also determined to modulate the host-cell immune reaction against numerous clinical strains of H1N1 and H3N2 influenza A virus [167]. The boosted IFN*β* gene expression with the TLR9/IRF7 pathway, observed following RSV treatment, advocated that IFN*β* likely acted synergistically with RSV to inhibit virus replication [168].

Finally, *in vivo* experiments showed that RSV increased disease-free survival and reduced viral pulmonary titers in mice infected with influenza A [161]. Notably, the effective dose of RSV was detected in both *in vitro* and *in vivo* findings with considerable variability. For instance, RSV's EC50 to many subtypes of *in vitro* influenza A and B viruses ranged from 5 to 26.3 *μ*g/mL, while RSV concentrations used to treat influenza-A-infected mice varied from 1 to 30 mg/Kg/day [165].

### 13.2. Respiratory Syncytial Virus

During the last years, the therapeutic potential of RSV against the respiratory syncytial virus has been studied because existing treatments have shown a controversial positive impact on clinical results, such as airway inflammation and AHR [169].

*In vitro* studies have indicated that RSV is a promising antiviral agent because it inhibits RSV replication and improves virus-associated airway inflammation and AHR by modulating host-cell signaling pathways associated with inflammation and lung injury [170]. In this respect, it has been detected that RSV inhibited virus-induced TIR-domain-containing adaptor-inducing interferon-*β* (TRIF) and TANK binding kinase one protein expression (TBK1), either by lessening the production of IL-6, the critical cytokine correlated with disease severity or diminishing INF*γ* levels via the expression of the sterile *α* and HEAT/Armadillo motif-containing protein (SARM) [171].

*In vivo* findings proved that the administration of RSV reduces the titer of the virus, the levels of IFN*γ*, and the number of inflammatory cells (e.g., NK cells, macrophages, and CD3+ T) in the lungs, thereby attenuating inflammation and hyperresponsiveness of the airways [172]. The levels of neurotrophins (nerve growth factor and brain-derived neurotrophic factor) involved in long-term inflammation associated with RSV infection have decreased following RSV therapy [173].

### 13.3. Other Respiratory Viruses

In *in vitro* research, SARS-CoV and MERS-CoV have lately been indicated to be responsive to RSV, indicating positive antiviral and anti-inflammatory effects [161]. Synthesized resveratrol derivatives have been shown to suppress SARS-CoV replication and reduce its clinical symptoms, while no resveratrol-derivative effects have been observed [174]. As for MERS-CoV, RSV was mentioned to lessen viral RNA expression and the infectious yield on such a dose-dependent basis (31.5–250 *μ*M and 150–250 *μ*M, respectively). Ironically, subsequent treatments with lower RSV concentrations (62.5 *μ*M) also prevented MERS-CoV replication [162]. This antiviral effect was responsible for decreasing virus-induced apoptosis, leading to boosted cell survival due to reduced caspase-3 levels in infected cells following RSV therapy [162].

Concerning HRV, RSV displayed higher dose-dependent antiviral activity versus reproduction in cultured cells and *ex vivo* nasal epithelia (therapeutic index: >111). After exposure to RSV, the activation of the inflammatory response in infected nasal epithelia was reversed [175]. RSV decreased IL-6, IL-8, and RANTES virus-induced secretion to levels close to those of uninfected nasal epithelia. RSV has also changed the expression of ICAM-1, the cellular HRV receptor, which is also functionally involved in inflammation [176]. Such outcomes may be of particular interest because the development of HRV-induced proinflammatory cytokines and chemokines in the pathogenicity for rhinovirus infection appears to be involving [177].

Eventually, RSV has also been shown to minimize dose-dependent hMPV replication (10–50 *μ*M) in airway epithelial cells with no effect on viral gene transcription and protein synthesis, suggesting that infected cell inhibition emerged viral assembly and/or release levels [178]. IL-8, RANTES, IL-1*α*, IL-6, TNF-*α*, CXCL10, and C\C Motif Chemokine Ligand-4 have substantially decreased the secretion of inflammatory mediators by modifying the expression of proinflammatory mediators (IL-8, RANTES, IL-1*α*, IL-6, TNF-*α*, CXCL10, and C\C Motif Chemokine Ligand-4) [179].

## 14. Functional Significance of Resveratrol

RSV has developed an essential interest in scientists and the public due to applauded health-beneficial effects [180]. Substantial research carried out *in vitro* and different animal models have reported potential upsides for human health following RSV administration, but these benefits remain recorded in human phase-3 studies [181]. While there is a significant lack of clinical indication for the applauded health advantages [98], RSV has increased considerable market traction as a dietary supplement [182]. The compound shows antimicrobial properties versus bacterial, fungal, and viral pathogens and the possible effects of RSV on several disorders, such as cancer [156]. As outlined in this analysis, RSV can inhibit bacterial and fungal growth, modify the expression of virulence elements, decrease biofilms' formation, decrease motility, and affect bacteria's susceptibility to various groups of traditional antibiotics [109]. RSV potentiates aminoglycosides' potency against many Gram-positive pathogens, and combinations would be evaluated *in vivo* to improve treatment efficacy [19]. Hence, it would be essential to examine whether RSV can have some applicability for aminoglycosides as a potentiator in animal models. By comparison, the effect of RSV consumption as a dietary supplement may also theoretically decrease the effectiveness of other groups of antibiotics such as fluoroquinolones, which needs more study in animal models [183]. Like so many other antimicrobial compounds, resistance problems can occur, and RSV enzymatic inactivation has been demonstrated as an example [184]. To determine the therapeutic potential of RSV as monotherapy or in combination with traditional antibiotics, research into appropriate animal models is greatly required.

There are currently a few medications available to treat respiratory viral infections, targeting the most popular etiological agents, such as influenza viruses and RSV, in particular. By comparison, there are currently no clinically approved antiviral drugs available for emerging respiratory virus pandemics such as SARS-CoV, MERS-CoV, and SARS-CoV-2 [185]. Given the insufficient antiviral treatments available for respiratory viruses and the nature of the clinical picture underlying the extreme complications associated with the virus, alternative possible therapeutic strategies have been explored to improve disease control.

## 15. Conclusions and Future Perspectives

Resveratrol, a natural agent, has gained significance in respiratory viral infections over the last few years for its therapeutic ability. As previously described, RSV and its analogs demonstrated antiviral activity versus infection viruses, RSV, HCoV, HRV, and hMPV, both using direct restraint of viral replication and host immune response modulation. Furthermore, anti-inflammatory as well as antioxidant activities, which underlie the cardioprotective impacts of RSV, can help alleviate the signs related to respiratory viruses' pathological symptoms.

A variety of issues, therefore, still need to be tackled. For instance, RSV dosing, capable of optimizing its health advantages without side effects, stays an area of comprehensive study. Nonetheless, as stated in advance, *in vivo* research studies examining RSV's antiviral activity applied various dosages and dosages periods, both of which reported a reduced viral load as the vital outcome. Besides, to provide a more detailed view, the review of RSV's pharmacokinetic profile ought to be conducted along with the research of its antiviral properties.

Given the issues mentioned above, additional data are required regarding the potential of RSV for the prevention and treatment of infectious disorders, particularly clinical experiments. Nonetheless, further efforts are needed for discovering new aspects of the utility of RSV. Future work should be directed towards that. It identifies a suitable synergistic combination for attaining a given outcome and also deciding if RSV may have additive or synergistic effects in conjunction with other treatments. RSV as a stand-alone therapeutic agent was the subject of the present research. But, it might be feasible that supplementation with RSV could synergistically enhance the effectiveness of therapeutic compounds. Ultimately, statistical analysis on the results of supplementation with long-term RSV is fundamental. RSV's acute effects are evident, but its action mechanism in long-term procedures is not yet apparent.

## Figures and Tables

**Table 1:** Review of the antimicrobial properties of resveratrol.

Samples	Strains	Methods	References
Resveratrol	*Cronobacter sakazakii Fec39*	Broth dilution	[96]
	*Cronobacter sakazakii MSDH*		[97]

Resveratrol	*Xylella fastidiosa Dixon Almond*	Agar dilution	[98, 99]
	*Xylella fastidiosa Tulare*		[100]

Resveratrol	*Pseudomonas aeruginosa ATCC 27853*	Disk diffusion, microdilution, and time-kill curves	[101]
	*Enterococcus faecalis ATCC 29212*		[98, 99]

Resveratrol extracted from wine	*Salmonella enterica ATCC 13076*	Time-kill curves	[102]
	*Escherichia coli ATCC 25922*		[103]

Resveratrol isolated from seeds of melinjo (*Gnetumgne mon* L.)	*Lactobacillus plantarum NRIC1067*	Agar dilution	[104]
	*Luconostoc mesenteroides 9a4*		[101]

Resveratrol isolated from grapes	*Helicobacter pyloriclinical strain G21, cagA negative*	Microdilution	[105, 106]
	*Helicobacter pyloriclinical strain 10K, cagA positive (cagA* ^*+*^)		[107]

Resveratrol	*Micrococcus luteus Presque Isle 456*	Disk diffusion and microdilution	[105, 106]
	*Streptococcus pneumoniae ATCC 6303*		[108]

Resveratrol	*Candida glabrata Y 33.90*	Agar dilution	[89]
	*Candida albicans SC 5314*		[64, 65]

Resveratrol	*Bacillus cereus*	Agar dilution and microdilution	[109]
	*Staphylococcus aureus*		[110]

Resveratrol	*Propionibacterium acnes ATCC 33179*	Broth dilution	[104]
	*Propionibacterium acnes ATCC 25746*		[111]

Resveratrol	*Candida albicans*	Microdilution	[91]
	*Enterococcus faecalis*		[97]

**Table 2:** Antimicrobial activity of resveratrol against bacteria and fungi.

Organisms	Identifiers	MIC (*μ*g/mL)	References
Gram-positive bacteria			
*Bacillus cereus*	ATCC 11778	50	[104]
*Enterococcus faecalis*	ATCC 29212	100	[98, 99]
*Enterococcus faecium*	D344R	128	[123]
*Mycobacterium tuberculosis*	H37Rv	100	[97]
*Streptococcus pyogenes*	Clinical isolate	*>*200	[26, 118]

Gram-negative bacteria			
*Pseudomonas aeruginosa*	ATCC 27853	*>*400	[101]
*Helicobacter pylori*	ATCC 43504	25	[117]
*Arcobacter cryaerophilus*	LMG 10829	50	[96]
*Neisseria gonorrhoeae*	Clinical isolate	75	[26, 118]
*Vibrio cholerae*	MCVO9	60	[110]
*Fusobacterium nucleatum*	ATCC 10953	100	[124]
*Campylobacter coli*	873	50	[119]

Fungi			
*Trichophyton mentagrophytes*	ATCC 18748	25–50	[125]
*Trichophyton tonsurans*	ATCC 28942	25–50	[125]
*Trichophyton rubrum*	ATCC 18762	25–50	[125]
*Epidermophyton floccosum*	ATCC 52066	25–50	[125]
*Microsporum gypseum*	ATCC 14683	25–50	[125]
*Candida albicans*	TIMM 1768	20	[112]
*Trichosporon beigelii*	KCTC 7077	10	[112]

**Table 3:** Mechanisms of resveratrol antibacterial activity.

Organisms	Mechanism descriptions	Concentration (*μ*g/mL)	References
Biofilm			
*Fusobacterium nucleatum*	Gene expression was downregulated in the biofilm	1.5625–25	[124]
*Escherichia coli*	Reduce expression of curli genes (csgA and csgB)	50–100	[103, 130]
*Propionibacterium acnes*	Reduction in biofilm production		[131]
*Vibrio cholerae*	Reduction in biofilm production	10–30	[110]
*Staphylococcus aureus*	No reduction in biofilm formation	20–100	[132]
*Staphylococcus aureus*	Repressed the *α*-hemolysin hla gene and the intercellular adhesion locus (icaA and icaD)	100	[98, 133]
*Arcobacter butzleri* and *Campylobacter* spp.	Reduction in biofilm production	12.5–50	[114]
*Listeria monocytogenes*	Reduction in biofilm production	50–100	[134]

Motility			
*Escherichia coli*	Reduction in swarming	20	[26]
*Proteus mirabilis*	Reduction in swarming	15–60	[135]
*Vibrio vulnificus*	Reduction in swarming	30 *μ*M	[136]

Quorum sensing (QS)			
*Yersinia enterocolitica*	Reduction in QS	10–20	[137, 138]
*Burkholderia* spp.	Reduction in QS	25 *μ*M	[139]

Toxins			
*Proteus mirabilis*	Inhibited and blocked swarming through an RsbA-dependent pathway	30–60	[135]
*Staphylococcus aureus*	Reduced haemolysis	20	[92, 133]
*Staphylococcus aureus*	Reduced haemolysis	10–100	[92, 133]
*Vibrio vulnificus*	Reduced toxin expression	10–30 *μ*M	[136]
*Vibrio cholerae*	Suppressed toxin activity	300–400 *μ*M	[140]

Adhesion			
*Vibrio vulnificus*	Reduced adhesion to host cells	10–30 *μ*M	[136]

Colonisation			
*Helicobacter pylori*	Decreased urease activity	6.25–400	[117]

## Data Availability

All data used in this study can be obtained by e-mail to the corresponding author.

## Abbreviations

RSV: Resveratrol
MICs: Minimum inhibitory concentrations
QS: Quorum sensing
TCSs: Two-component systems
CT: Cholera toxin
ROS: Reactive oxygen species
PKC: Protein kinase C
TRIF: TIR-domain-containing adaptor-inducing interferon-*β*
TBK1: TANK binding kinase 1.
